# Evaluation of attitude to, knowledge of and barriers toward research among medical science students

**DOI:** 10.1186/s12930-015-0019-2

**Published:** 2015-02-11

**Authors:** Mahtab Memarpour, Ali Poostforoush Fard, Roghieh Ghasemi

**Affiliations:** Department of Pediatric Dentistry, School of Dentistry, Shiraz University of Medical Sciences, Shiraz, Iran; Department of Epidemiology, School of Health, Shiraz University of Medical Sciences, Shiraz, Iran; Student Research Committee, School of Dentistry, Shiraz University of Medical Sciences, Shiraz, Iran

**Keywords:** Approach, Awareness, Medical students, Investigate, Research

## Abstract

**Background:**

Plans to increase the role of students in health research require data on students’ knowledge and views of research. The aim of the study was to evaluate these factors toward research among medical science students.

**Methods:**

Undergraduate and postgraduate students of three medicine, dentistry and pharmacy schools in Shiraz were enrolled in a cross-sectional descriptive study using questionnaires to provide details of the parameters of attitude to, knowledge of and barriers toward research for each individual. All data was coded for each of the parameters. Data analyses were performed by one-way ANOVA/Tukey and Student’s t, Pearson’s correlation and Chi-squared tests.

**Results:**

A total of 384 questionnaires were returned complete. Mean student scores for attitude, knowledge and barriers were 68.97 ± 12.56, 70.99 ± 20.97 and 75.27 ± 15.38, respectively. On the knowledge parameter, 77.8% of students’ scores fell above the middle of the possible attainable score, but 90% of attitude scores came in at below the middle of the possible attainable score. Undergraduate students (70.27 ± 12.00) showed a more positive attitude to research than postgraduate students (65.57 ± 13.06) (p = 0.001). Female students (72.97 ± 20.54) had greater knowledge than males (67.09 ± 21.56) (p = 0.010). Many barriers were highlighted by students such as lack of funding support and lack of time for research.

**Conclusions:**

Students showed favorable knowledge of research, but their attitude to the field was inadequate. More attention must be placed on these parameters in the curriculum to improve student interest in health research. The impact of barrier factors on research demonstrates that there is a need for greater availability of information in order to solve the problems and change strategies for research.

**Electronic supplementary material:**

The online version of this article (doi:10.1186/s12930-015-0019-2) contains supplementary material, which is available to authorized users.

## Introduction

In the present day, one of the best measures of scientific progress in a country is the research situation in their scientific communities [1]. Therefore, the concern over conducting scientific and accurate research has increased in most countries, both industrial and developing. This trend may be due to the desire to resolve the health care problems in their communities, to establish independence from other countries or to compete with them.

Research is a systematic process to achieve new knowledge, science or invention by the use standard methods [2]. Health research has an impact on the prevention, diagnosis and treatment of diseases and especially on health care programs policy [3]. Insufficient attention to research by a government and the educated members of a community may contribute to scientific and knowledge lag within the national community but also in the world as a whole [4]. Sometimes the trend in research is favoured by educated members, while the shortfall in basic and valuable research may reflect other factors that have influence on the research. The three main factors seen to impact on research success in the literature are: attitude to, knowledge of and barriers toward research [5-14].

One of the most important factors underlying any study is the researchers’ beliefs, as it is these that motivate them to undertake a study in the first place [4]. The attitude to health research stems from the researchers’ curiosity and interest in a particular subject or their wish to solve a problem within a community [5-8]. Performance of research fitted to the health needs of each society should be encouraged for consideration by their own educated sectors. The literature reports positive attitudes toward research among the majority of Irish [11], Pakistani [5,7,9], Croatian [10] and New Zealand [12] medical students throughout their career.

Adequate knowledge of the study subject and awareness of research principles are essential prerequisites for any study. Some previous studies of medical students showed that they had inadequate knowledge of the scientific inquiry process, but that they were nonetheless interested in pursuing research in the future [5,6,10].

The final factor directly affecting the performance of research lies in the barriers against researchers. The main parameters reported in the literature as barriers to research among medical students included: inadequate knowledge of study design or interpretation of study results, time limitations [11,13-15] and restrictions in funding support [9,14-16]. Other factors mentioned as barriers include: lack of research training [1,9], uncertainty about the ability to successfully complete a study (lack of research self-efficacy) [1,11,14,15], little support from mentorship [11,14,17,18], lack of interest in research [19] and limited access to data sources (i.e. internet), materials and equipment [6].

Given the role of research in health care programs and the fact that few studies are available on the importance of research among medical science students, the aim of this study was the “evaluation of attitude to, knowledge of and barriers toward research among students of medicine, dentistry and pharmacy studying at Shiraz University of Medical Sciences 2012-2013”. The results will be used as the basis of recommendations and a strategy to improve research among medical science students in Shiraz.

## Methods

### Subjects

The research protocol for this cross-sectional study was submitted to the Human Ethics Review Committee of the Faculty of Dentistry Shiraz University of Medical Sciences. Following approval from the Committee, 410 students were enrolled in the study. Of the 216 medical students, 135 agreed to participate in the study, along with 144 out of 151 of dental students and 131 of the 152 pharmacy students initially enrolled in the study. The cohort included all of the undergraduate students enrolled in the last two years of their respective medical science schools (5^th^ and 6^th^ years) in Shiraz, plus postgraduate students in all three fields regardless of the year of their education. Exclusion criteria for the study included: unwillingness to participate in the survey, undergraduate students from below the 5th year of their course and any responses where no more than three questions were answered.

### Questionnaire

A 3-page, self-reporting questionnaire was distributed to the students by one researcher who explained the aim of the study, and those students who showed an interest in participating were able to consult the surveyor who remained present to answer any questions raised by respondents while they were completing the forms. The questionnaires were distributed directly to individual students and were collected immediately after completion. Both undergraduate and postgraduate students in the three fields of medical sciences (medicine, dentistry and pharmacy) were included in the study. The definition of postgraduate student includes: medical residents (involving those in the clinical practice, but not PhD students involved in basic sciences), all the dental resident students and all PhD pharmacy attending in the pharmacy. All participants were assured that their responses would remain secret.

The questions included in the questionnaire were obtained on the basis of a comprehensive literature review [9,10,14,15,20-22]. The content of the questionnaire was adapted from previous studies with efforts made to make the questionnaires appropriate to our local university. The questionnaire included three main sections to evaluate student views on attitudes to, knowledge of and barriers towards research (Additional file 1). The questionnaire addressed:

1. Demographic information such as: age, gender, marital status, field of study, level of education, participation in research projects.
2. Attitudes towards research were assessed by 27 questions. The answers were evaluated by 5-point Likert rating scale ranging from strongly agree (score 1) to strongly disagree (score 5). The total of attitude scores as well as barrier scores for each student were computed as a sum of the total number scores (5-point Likert) answered for the questions. The questions were centered on the perceived role of research in their education, life and future career. Some questions also raised discussion of the importance of research class, the kind of research preference, reasons for having interest in the research and plans to participate in research.
3. Knowledge of research was investigated through 8 questions to evaluate basic and preliminary knowledge of different kinds of research studies, statistics, scientific writing, database resources. Each correct response earned a score of 1 and each incorrect answer received a score of 0.
4. Barriers toward research were assessed through 32 questions and evaluated in the same way as described for assessment of attitude. The questions concerned limitation factors on research such as: inadequate financial support, problems in performing research (i.e.; lack of access to equipment and research materials), lack of time, inadequate motivation, inadequate mentor support and acknowledgement of researchers.

The relevance of the questions and the comprehensibility of the questionnaire were assessed by a panel of 10 professors and 25 students in each of the institutes prior to implementation in order to ensure there was no difficulty in understanding and responding to the questions. The reliability coefficient accessed by Cronbach alpha was 0.75 for attitude, 0.88 for barriers and 0.71 for knowledge.

### Analysis

#### Statistical analysis

All of the data from the completed forms was collected and coded for each of these parameters to assess the medical, dental and pharmacy students’ responses to the three fields of research, including the undergraduate and post graduate students. Also the respondents were compared according to field of education as well as level of education. The students’ answers were compared to each other to identify any impact of age, sex, marital status or level of education on their responses. The quantitative variables were presented as percentages and the qualitative data was presented as means and standard deviations of variables relating to the level and field of education.

Data analyses were performed by means of the SPSS for Windows version 15.0 statistical package. One-way ANOVA/Tukey and Student’s t tests were used to compare the mean scores and ages in different levels and field of education, respectively. Pearson’s correlation coefficient was used to assess the relationship between age and scores. The chi-square test was employed to draw comparisons between groups on a basis of sex and marital status. Of the questionnaires collected, any with more than two missing responses were discarded. Throughout the study p < 0.05 was considered statistically significant.

## Results

Completed forms were received from 384 undergraduate and postgraduate students. Reponses were received from 276 undergraduates (mean age 23.75 ± 1.60) and 108 postgraduates (mean age 27.85 ± 3.06). In total 66.4% were women and 33.6% were men. All of the students surveyed had participated at least in one piece of research, but only 36.8% had published their work (31.2% of undergraduate students and 42.4% of postgraduate students). Table 1 shows demographic characteristics of participants.

**Table 1:** **Demographic data of students participating in the current study**

		**n**	**Undergraduate students**	**Postgraduate students**
**Sex**	Male	130	82 (29.8%)	48 (43/4)
	Female	254	194 (70.2%)	60 (56/6%)
**Marital status**	Single	296	230 (83/6%)	66 (61/7%)
	Couple	88	46 (16/4%)	42 (38/3%)
**Field of education**	Medical	121	85 (70/2%)	36 (29/8%)
	Dental	138	101 (73/4%)	37(26/6%)
	Pharmacy	125	90 (73/2%)	35 (26/8%)

The overall mean scores of students on attitude, knowledge and barriers were 68.97 ± 12.56, 70.99 ± 20.97 and 75.27 ± 15.38, respectively. Female students (72.97 ± 20.54) had greater knowledge than males (67.09 ± 21.56) (p=0.010), and single students (69.73 ± 12.37) had better attitude than their married peers (66.12 ± 12.54) (P = 0.020). The age of students was significantly correlated with both knowledge (r = -0.102, p = 0.048) and attitude scores (r = -0.170, p = 0.001) in an inverse direction.

Comparisons between the 3 different schools regardless of level of education showed a mean for knowledge in medical students significantly lower than that of pharmacy and dentistry students (p < 0.001, p = 0.012) respectively. The mean attitude score of undergraduate students (70.27 ± 12.00) was significantly greater than that of postgraduate students (65.57 ± 13.06) (p = 0.001). However, there was no significant difference between the education levels of students in terms of knowledge and barrier scores (p = 0.974, p = 0.791, respectively). Table 2 shows comparative means and standard deviations between on research subjects between sex and marital status groups as well as field and level of education groups.

**Table 2:** Comparison of mean (±SD) of research subjects for demographic characteristics

**Variable**		**Attitude**	**Knowledge**	**Barrier**
**Sex**	Male	69.86 ± 12.47	67.09 ± 21.56	75.79 ± 14.57
	Female	69.58 ± 12.48	72.97 ± 20.54	74.78 ± 15.82
**P value**		0.010*	0.354	0.542
**Marital status**	Single	69.73 ± 12.37	70.90 ± 20.26	74.98 ± 15.23
	Couple	66.12 ± 12.54	71.51 ± 23.61	76.25 ± 15.72
**P value**		0.020*	0.814	0.507
**Field of education**	Medical	70.90 ± 5.04^a^	72.36 ± 18.47^a^	75.14 ± 16.14^a^
	Dental	67.84 ± 11.39^a^	64.77 ± 22.36^b^	74.61 ± 13.4^a^
	Pharmacy	68.23 ± 0.82^a^	75.18 ± 20.69^a^	76.13 ± 16.7^a^
**P value**		0.106	<0.001*	0.729
**Level of education**	Undergraduate students	70.27 ± 12.00	75.18 ± 14.56	71.24 ± 20.88
	Postgraduate students	65.57 ± 13.06	75.24 ± 17.35	70.60 ± 21.36
**P value**		0.001*	0.974	0.791

In the case of field of education, different letters in each column show significant differences between the fields (Tukey HSD test).

p < 0.05 is significant*.

**Figure 1:**
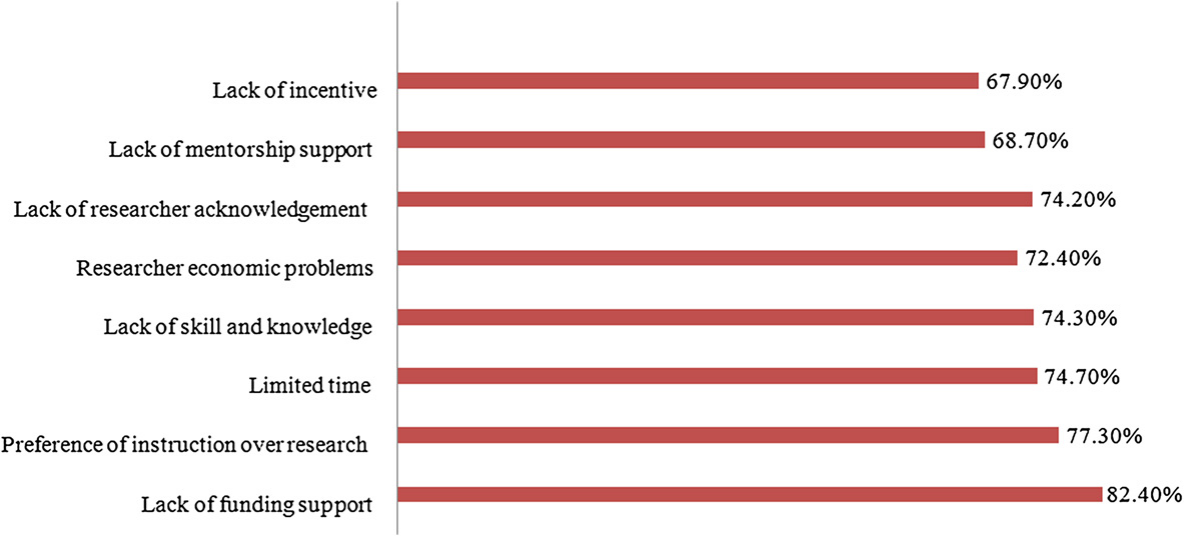
**Percentage of main barriers toward research by medical students.**

Table 3 shows comparative means and standard deviations between field and level of education on research subjects. The levels for each of the 3 threads: attitude, knowledge and barriers to health research, were evaluated by the percentage of students falling in the quartiles of the possible score for each field (Table 4). Data showed that most of student (90.5%) had an attitude score to research which was lower than half of possible attainable score, although 77.9% fell above half of possible attainable score on the knowledge parameter and showed adequate knowledge. A large number of students (66.7%) were more interested in clinical research than the other forms of research such as social and in vitro study (45.9%). Inadequate financial support was cited as the main barrier, followed by a preference for academic instruction over research, limited time and lack of research skills and knowledge. Figure 1 shows the highest agreement percentage of respondents in the various fields of barriers.

**Table 3:** **Comparison of mean (±SD) of research subjects for educational characteristics**

**Field**	**Educational level**	**Attitude**	**P value**	**Knowledge**	**P value**	**Barriers**	**P value**
**Medical**	UGS	70.4 ± 14.85	0.244	5.11 ± 1.80	0.547	76.43 ± 14.89	0.179
	PGS	68.52 ± 15.39		5.33 ± 1.75		72.11 ± 18.65	
**Dental**	UGS	69.28 ± 10.62	0.017	6.088 ± 1.59	0.384	74.03 ± 13.34	0.411
	PGS	64.55 ± 12.59		5.81 ± 1.82		5.69 ± 1.51	
**Pharmacy**	UGS	69.91 ± 10.40	0.070	5.82 ± 1.47	0.679	74.54 ± 15.63	0.525
	PGS	63.96 ± 10.83		5.69 ± 1.51		5.69 ± 1.51	

UGS: undergraduate students, PGS: postgraduate students.

p < 0.05 is significant*.

**Table 4:** **Distribution of attitude, knowledge, and barrier scores of participants regarding the percentages of total attainable score**

**Percent of total score**	**Attitude**	**Knowledge**	**Barriers**
**<25%**	49 (13.2%)	21 (5.5%)	97 (25.7%)
**26%-50%**	286 (77.3%)	64 (16.7%)	260 (69.0%)
**51%-75%**	34 (9.2%)	168 (43.8%)	19 (5%)
**>75%**	1 (0.3%)	131 (34.1%)	1 (0.3%)

## Discussion

In the present day, a global approach to scientific studies has developed in medical education which leads to increased number of articles being published throughout the world.

In the present study, the questionnaires were devised on the basis of a comprehensive literature review [9,10,14,15,20-22]. We are aware of the limitations of evaluations based on the use of self-reporting, as used in our study, but we consider the method useful in preliminary evaluation of the views of medical science students to research in the various parameters of attitude, knowledge and barriers. Our study did not attempt to assess the sort of research performed by students, but we are aware that the results of any such evaluation can be useful to open new perspectives on research and the revision of curricula.

In the present study most of the students (90%) showed an attitude to research that fell below the 50% level, whereas Vodopivec et al. [10] and Amin et al. [6] reported a positive attitude to research among medical students in Croatia and the Arab Universities. Khan et al. also reported moderate attitude to research among Pakistani medical students [5,8]. These differences may be related to differences between countries and students, and the impact of other factors such as barriers may have a heavy influence on levels of interest in research. The range covered by the questions in our comprehensive questionnaire and the detail given in the responses may also account for some of this variation. The current study revealed a better attitude to research among undergraduate than postgraduate students, and increasing age and level of education as well as marriage, were clearly seen to have an adverse effect on attitude to and knowledge of research. This may be due to the heavy workload of postgraduate students in their research and studies coupled with marital responsibilities [9] and the belief that research will have little role in their future career [5]. The findings of Askew et al. were similar to ours, reporting that younger physicians were more enthusiastic about research [23]. Khan et al. also reported that residents’ knowledge and attitudes towards research did not improve significantly with their increasing years of education [5], however; Vukaklija et al. reported the opposite, as they found the attitude toward research improved among undergraduate students with each increasing year of education [1]. This particular difference may correlated to the level of students evaluated (undergraduate or postgraduate) or variations according to the field of study, also bearing in mind that barriers to research may vary according to the individual situation in each country.

The current study showed that female students had greater knowledge than their male peers, but attitude to research did not differ between the sexes. Similar results were obtained by Amin el al. [6]. In contrast to our study, however, studies in Pakistan and the USA revealed male medical students showed a better attitude to research than their female peers [5,8,9,24,25]. The differences may be related to data collection from different populations, variations in sample size [9] and the increasing of acceptance female students in our medical universities.

Our study showed that all of the students surveyed were involved in a research project. This may be due to the mandatory thesis course for graduation in the last years of scientific education, and a similar policy appears to be implemented in many universities [6,9]. Considerations of research in the curriculum varies across countries and universities [14,26]. Pruskil et al. [27] showed that the reformed curricula led to increased student involvement in research activity. Vujaklija el al. [1], Khan et al. [5] and Wang and Guo [15] stated that assessed projects and mandatory research improve experience and training in research, have a positive impact on students and motivate them to undertake further research in the future [8,17].

Lack of research activity may be due to inadequate researcher knowledge [28]. Present study showed students’ knowledge was moderately favorable. Also, our data showed the students had poor awareness of statistical techniques, the essentials for writing articles. Similar inadequate knowledge of these items has been mentioned before in the literature [6,28,29]. Our results showed that the mean for knowledge in medical students was significantly lower than that of pharmacy and dentistry students, which may partly be due to the heavy workload in hospitals and the limited time available to participate in the research classes [5,6,8,9,11,13,14,19,26].

In the current study, the main barrier to research was reported as inadequate financial support followed by the preference for academic instruction over research, limited time and lack of research skills. In addition, the lack of researcher motivation or acknowledgment, researcher economic problems associated with the lack of payment for research, and lack of mentorship were considered as barriers by students. The same results have also been reported in other studies [6,9,11,26,30].

Just as in our study, lack of research funding support was also cited as the main barrier for students in previous studies [5,9,13]. This may be related to the fact that limits on funding for research involving expensive materials and equipment in some countries lead to a low incentive for research [5]. Institutes should try to seek funding support from resources other than the government and they should encourage researchers to seek out grants and awards [14].

Limited time due to heavy workload was commonly reported in previous studies [5,6,8,9,11,13,14,19,26], with this experience noted especially by residents who become engrossed in clinical practice that engages them physically and mentally to such an extent that they are left with no free time for research [5,6]. Setting aside a specific time slot for research activity in the student curriculum may be helpful in reducing this barrier [26].

The role of faculty staff in teaching research principles to students has been noted in some studies [1,5,6]. Professors can create positive awareness of research in students [31] and can guide students to become aware of health problems within their society, using the approach to help solve problems [6]. Lack of support through mentorship and the reduced effectiveness of research has already been reported in the literature [6,24,14]. Poor guidance by professors may lead to confusion amongst students during study stages [6,11,26] lead to student dissatisfaction [21,31] and lack of financial support when problems are encountered [6,11,26]. It is strongly recommended that professors be encouraged to participate as active mentors involved in all stages of the study [6].

## Conclusions

Medical science students in three schools of medicine, dentistry and pharmacy showed a favorable knowledge of research, but their attitude toward the process ranked at below moderate. Undergraduate and single students showed a better attitude than residents. Females had a better knowledge of research than males. The majority of students considered there were barriers to the performance of research. While all students were involved in at least one research project, students are given no mandatory input on research theory and practice that might deepen their understanding of the research process.

## Additional file

Additional file 1:
**The questionnaire of attitude to, knowledge of and barriers towards research.**
