# Supplementary material for: Evaluation of attitude to, knowledge of and barriers toward research among medical science students
Source: Asia Pac Fam Med. 2015 Feb 11;14(1):1. doi: 10.1186/s12930-015-0019-2 (PMC4336721; doi:10.1186/s12930-015-0019-2)
Supplement: Additional file 1: — The questionnaire of attitude to, knowledge of and barriers towards research. [file 12930_2015_19_MOESM1_ESM.docx]

**Additional file 1: The questionnaire of attitude to, knowledge of and barriers towards research.**

| Dear Colleague,  The following questionnaire is designed to help our researchers to “Evaluate attitude to, knowledge of and barriers toward research among medical science students”. We would be grateful if you would help us by completing the form. |
| --- |
| **Student demographic information** |
| -Gender: female male -Marital status: single married - Year of birth - School of education:  Medical, Dental , Pharmacy  -Entry year:……. -Undergraduate student , -Postgraduate student  |

| **Totally disagree** | **Disagree** | **No comment** | **Agree** | **Totally agree** | **Student attitude toward research & assessment approach** |
| --- | --- | --- | --- | --- | --- |
|  |  |  |  |  | 1- Science gives us have better understanding of the world |
|  |  |  |  |  | 2- Every physician ,dentist, pharmacist should be familiar with the scientific method |
|  |  |  |  |  | 3- Knowledge is necessary to achieve true results from scientific methods |
|  |  |  |  |  | 4- We have a healthier life with less discomfort (or worry) with science |
|  |  |  |  |  | 5-Thinking about scientific methods is dull and boring |
|  |  |  |  |  | 6- I trust the research results reported to the public |
|  |  |  |  |  | 7-Each student should do research on their course, even if it is not in the training program |
|  |  |  |  |  | 8-Medical students can design & perform a research project and write scientific paper |
|  |  |  |  |  | 9- I like to participate in research (whether or not it is compulsory such as dissertation) |
|  |  |  |  |  | 10-I tend to perform clinical research |
|  |  |  |  |  | 11- I tend to perform research with the community |
|  |  |  |  |  | 12- I tend to do laboratory studies related to clinical practice |
|  |  |  |  |  | 13- Performing research is important for me to become a specialist |
|  |  |  |  |  | 14-I plan to use research as part of my professional work |
|  |  |  |  |  | 15- Skills that I gain during research are useful in my future work |
|  |  |  |  |  | 16- Taking time to research is time wasted, if it doesn’t enhance my future career |
|  |  |  |  |  | 17- Direct thinking and reflection on research plays an important role in my life every day |
|  |  |  |  |  | 18- Research should be offered in training to all students in studies classes |
|  |  |  |  |  | 19- I will be glad to participate in research classes (consent to participate in the class) |
|  |  |  |  |  | 20- I would like to replace another class related to my field with a research class |
|  |  |  |  |  | 21- Education on research should be compulsory in the student curriculum |
|  |  |  |  |  | 22- Doctoral students should be involved in research during their courses |
|  |  |  |  |  | 23- Research is beneficial, because it improves critical thinking |
|  |  |  |  |  | 24- Research is useful, because it helps to change policy & policies |
|  |  |  |  |  | 25- Impact factor is the most important issue in selecting which journal to target with our article |
|  |  |  |  |  | 26- I wish to publish the results of some research |
|  |  |  |  |  | 27- Research methodology workshops at the university were very effective for me |

| **Totally disagree** | **Disagree** | **No comment** | **Agree** | **Totally agree** | **Assess the barriers to research students** |
| --- | --- | --- | --- | --- | --- |
|  |  |  |  |  | 28- Lack of timely funding of research & lack of funds |
|  |  |  |  |  | 29- Lack of appropriate databases |
|  |  |  |  |  | 30- Lack of access to laboratory equipment for performing research project |
|  |  |  |  |  | 31- Lack of access to studies across the country |
|  |  |  |  |  | 32- Distribution of provider center for research, lack of cooperation between research centers |
|  |  |  |  |  | 33- Lack of suitable research space |
|  |  |  |  |  | 34- Dissatisfaction with encouragement of researchers to do research |
|  |  |  |  |  | 35- Poor attention given to researchers and creative faculty |
|  |  |  |  |  | 36- Priority on education over research in university |
|  |  |  |  |  | 37- Lack of time to do research because of educational tasks |
|  |  |  |  |  | 38- Poor collaboration between departments and research centers |
|  |  |  |  |  | 39- Personal economic & financial problems |
|  |  |  |  |  | 40- Insufficient research skills |
|  |  |  |  |  | 41- Lack of familiarity with research studies |
|  |  |  |  |  | 42- Lack of familiarity with statistical analysis |
|  |  |  |  |  | 43- Lack of skills for writing papers |
|  |  |  |  |  | 44- Lack of skills for submitting articles |
|  |  |  |  |  | 45- Lack of familiarity with research proposal writing |
|  |  |  |  |  | 46- Lack of good research ideas |
|  |  |  |  |  | 47- Lack of ability to publish article after the completion of the project |
|  |  |  |  |  | 48- Boring and difficult to research because of the lack of skill |
|  |  |  |  |  | 49- Lack of research needs and priorities in university health system |
|  |  |  |  |  | 50- Lack of coordination of research priorities with research ideas |
|  |  |  |  |  | 51- Inappropriate or insufficient consultation before drafting research proposals |
|  |  |  |  |  | 52- Inappropriate or insufficient guidance for writing |
|  |  |  |  |  | 53- Lack of research applications in personal life and professional job |
|  |  |  |  |  | 54- Lack of interest in research |
|  |  |  |  |  | 55- Lack of significant income to do research |
|  |  |  |  |  | 56- Lack of confidence in potential for completing research |
|  |  |  |  |  | 57- Prefer to use the free time to do other task |
|  |  |  |  |  | 58- Fear of making mistakes in research and being blamed by others |
|  |  |  |  |  | 59- Lack of professor input with students (research undertaken by full-time students) |

| **Assessment of student knowledge of research** |
| --- |
| **60-A scale from 1 to 5 (like grades on the examination) is called:**  A) Ratio scale B) Ordinal  C) Nominal D) Interval |
| **61-Which following software is used for reference of science article?**  A)SPSS B)Access  C)Concept Map D)End Note |
| **62-What is the blood type of a variable scale?**  A)Nominal B)Relative  C)Distance D)Serial |
| **63-Which way of writing reference is approved and for writing general medical dissertations and most medical journals?**  A)Vancouver B)Harvard C)None D)Chicago |
| **64-What is the definition of Medline?**  A) The first and best known on-line medical journals  B) Association for informed medical providers  C) Print format chosen medical  D) Medical database |
| **65-In which part of an article do you talk about the study limitations?**  A) Acknowledgment B) Methods and Materials  C) Discussion D) Introduction |
| **66-Which of the following types of research sees sample loss more commonly**  A) Clinical trial B) Sectional  C) Case – control D) Cohort |
| **67-Which item is not part of a scientific original paper?**   1. Discussion B) Introduction   C) Letters to the Editor D) Methods and Materials |
